# Probing the stability of the “naked” mucin-like domain of human α-dystroglycan

**DOI:** 10.1186/1471-2091-14-15

**Published:** 2013-07-01

**Authors:** Manuela Bozzi, Enrico Di Stasio, Giovanni Luca Scaglione, Claudia Desiderio, Claudia Martelli, Bruno Giardina, Francesca Sciandra, Andrea Brancaccio

**Affiliations:** 1 Istituto di Biochimica e Biochimica Clinica, Università Cattolica del Sacro Cuore, Largo F. Vito 1, Roma, 00168, Italy; 2 Istituto di Chimica del Riconoscimento Molecolare (CNR) c/o Istituto di Biochimica e Biochimica Clinica, Università Cattolica del Sacro Cuore, Largo F. Vito 1, Roma, 00168, Italy

**Keywords:** Dystroglycan, Dynamic light scattering, Capillary electrophoresis, Mass spectrometry

## Abstract

**Background:**

α-Dystroglycan (α-DG) is heavily glycosylated within its central mucin-like domain. The glycosylation shell of α-dystroglycan is known to largely influence its functional properties toward extracellular ligands. The structural features of this α-dystroglycan domain have been poorly studied so far. For the first time, we have attempted a recombinant expression approach in *E. coli* cells, in order to analyze by biochemical and biophysical techniques this important domain of the α-dystroglycan core protein.

**Results:**

We expressed the recombinant mucin-like domain of human α-dystroglycan in *E. coli* cells, and purified it as a soluble peptide of 174 aa. A cleavage event, that progressively emerges under repeated cycles of freeze/thaw, occurs at the carboxy side of Arg461, liberating a 151 aa fragment as revealed by mass spectrometry analysis. The mucin-like peptide lacks any particular fold, as confirmed by its hydrodynamic properties and its fluorescence behavior under guanidine hydrochloride denaturation. Dynamic light scattering has been used to demonstrate that this mucin-like peptide is arranged in a conformation that is prone to aggregation at room temperature, with a melting temperature of ~40°C, which indicates a pronounced instability. Such a conclusion has been corroborated by trypsin limited proteolysis, upon which the protein has been fully degraded in less than 60 min.

**Conclusions:**

Our analysis indirectly confirms the idea that the mucin-like domain of α-dystroglycan needs to be extensively glycosylated in order to reach a stable conformation. The absence/reduction of glycosylation by itself may greatly reduce the stability of the dystroglycan complex. Although an altered pattern of α-dystroglycan O-mannosylation, that is not significantly changing its overall glycosylation fraction, represents the primary molecular clue behind currently known dystroglycanopathies, it cannot be ruled out that still unidentified forms of αDG-related dystrophy might originate by a more substantial reduction of α-dystroglycan glycosylation and by its consequent destabilization.

## Background

α-Dystroglycan (DG) is the peripheral subunit of the DG complex that lies at the basement membrane/sarcolemma cellular crossroad and that is important for muscle fibers’ stability. α-DG glycosylation pattern is considered crucial for its function in terms of laminin binding affinity, and a disruption of the α-DG/laminin network is at the basis of a group of diseases spanning from the severe congenital to the much milder limb-girdle muscular dystrophies, defined as secondary dystroglycanopathies [1].

The DG complex is composed of two subunits (α and β) that are liberated upon an early processing step of a unique precursor of 895 aa in humans (pre-DG), that is thought to take place within the endoplasmic reticulum right after translation. The α-DG is an extracellular protein, highly and heterogenously glycosylated, whilst the β-DG subunit is transmembrane. A biochemical analysis of the α-DG subunit reveals a structural organization characterized by two terminal globular portions separated by an elongated central section (mucin-like) [2]. Although a few glycosylation sites have been proposed and/or localized within the two globular domains of α-DG, most of the O-linked oligasaccharidic chains protrude from Thr and Ser residues placed within the central mucin-like domain [3].

The whole maturation process of α-DG is known to be highly regulated and to take place in the typical subcellular locations, where multiple enzymes are required for the post-translational modification of the α-DG core protein [4]. An ever growing list of acclaimed, putative or uncharacterized glycosyltransferases (see [5] and references therein) is thought to be involved in the decoration of α-DG along the endoplasmic reticulum and the Golgi apparatus. It is quite unlikely that α-DG would represent the unique target of such a large number of enzymes [6]. Nevertheless, a long list of *nonsense* or *missense* mutations affecting these enzymes seems to lead to muscular dystrophy phenotypes whose major molecular trait is believed to be a reduction of α-DG functionality, depending on the alteration of its glycosylation shell. The general concept is that a hypoglycosylated α-DG would be less active in binding laminin-2, or other matrix binding partners, thus provoking a reduction of the overall stability of the sarcolemma preceding the onset of inflammation, fibrosis and necrosis typical of many severe muscular dystrophies.

On such premise, it is rather clear that the mucin-like central section of α-DG represents its most interesting functional hot spot. Recently, several works have highlighted specific Thr (and Ser) residues as glycosylation sites and/or identified the structure of the modifying saccharides [7-12]. However, three-dimensional structures referring to this specific domain of α-DG alone or in complex with laminin or other ligands are not available yet.

An approach that has been particularly successful in our lab for the study of the subdomain organization of α- and β-DG subunits, is represented by the expression of unmodified recombinant peptides spanning portions or isolated subdomains of both the DG subunits [1]. Therefore, we have attempted a similar approach for the study of the mucin-like portion of α-DG. We have been able to obtain reasonable amounts of a recombinant protein spanning the entire mucin-like portion of human α-DG in a soluble fashion, and we have characterized its conformation with a series of biophysical techniques. Such recombinant peptide may be useful and pave the way for further biochemical and biotechnological studies on this important domain of α-DG.

## Results and discussion

The mucin-like domain cannot be quantitatively obtained in a glycosylated fashion from eukaryotic cells and therefore we decided to apply our *E. coli*-based recombinant approach already used for the expression and purification of other DG domains [1,13]. The primary structure of the portion of human α-DG under analysis, including the Arg-Val site that we have found to be sensitive to proteases (see below), is reported in Additional file 1: Table S1.

**Figure 1:**
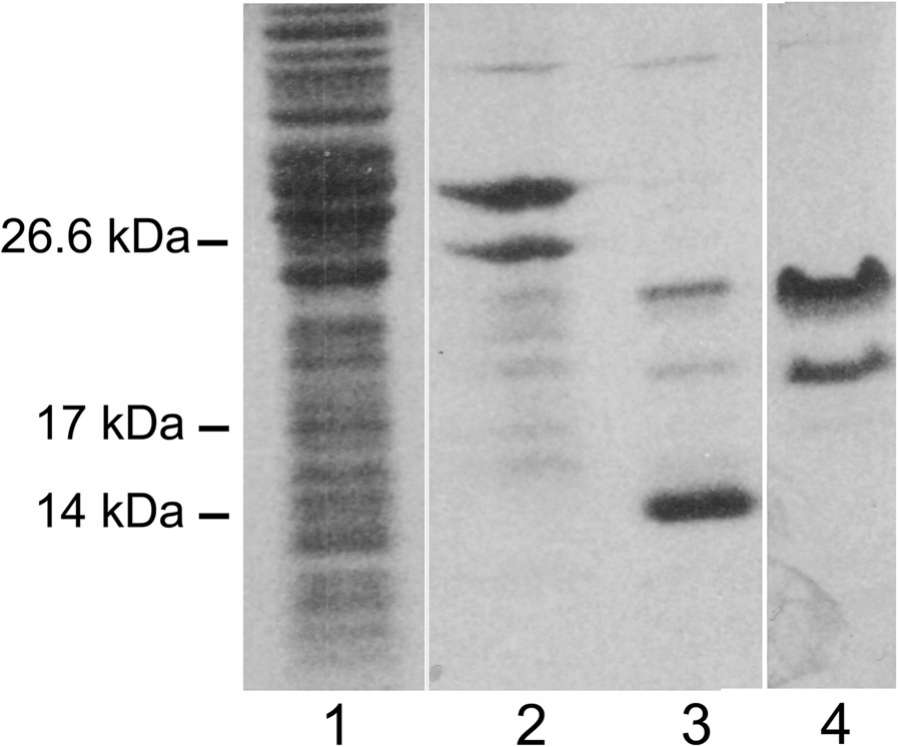
**Purification of the recombinant α-DG mucin-like domain, α-DG(316–484).** α-DG-DG(316–484) purification steps were analysed by 12% SDS-PAGE stained with Coomassie Brilliant Blue R-250 dye. Lane 1, overall cell lysate from *E. coli* expressing the chimaeric protein Trx-α-DG(316–484); lane 2, purified Trx-α-DG(316–484); lane 3, Trx-α-DG(316–484) upon thrombin cleavage: two upper bands referring to α-DG peptides plus a lower band of Trx are observable; lane 4, purified and concentrated α-DG(316–484). The lower band refers to a processed α-DG(316–461) product that has been characterized by mass spectrometry.

Samples collected after each step of the expression and purification protocol were separated by SDS-PAGE (Figure 1). It is noteworthy that the electrophoretic mobility of the purified mucin-like domain, α-DG(316–484), is much lower than expected on the basis of its molecular mass as reported for natively unfolded proteins [14].

The protein yield is good (circa 5 mg/l of culture), and the full-length protein is relatively stable, since it can be submitted to thrombin digestion and retrieved in a thioredoxin-free form. In addition, the protein remains soluble during all the purification steps. Nevertheless, it is evident a C-terminal processing event (see the lower band in lanes 2 and 4 of Figure 1) that is likely to take place within the *E. coli* cells, and that we have also observed after repeated cycles of freezing and thawing (data not shown).

**Figure 2:**
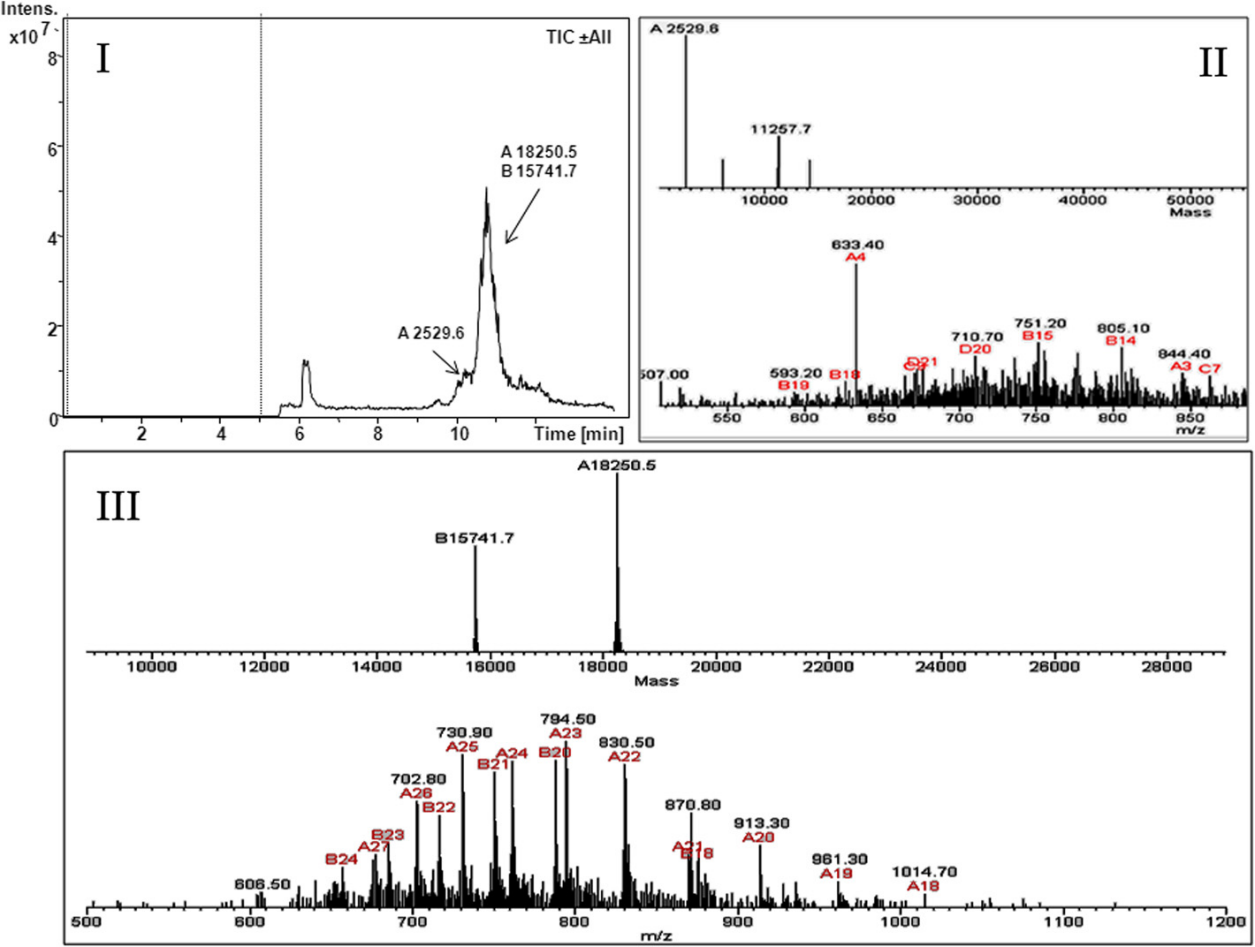
**Capillary electrophoresis-mass spectrometry (CE-MS) experiments.** Total Ion Current (TIC) plot of the purified fraction of human recombinant mucin-like domain, α-DG(316–484), obtained by CE-ESI-IT-MS analysis (panel **I**). The average MS spectra recorded in the main peak (panel **III**) and in its front part (panel **II**) with their relative deconvolution are also shown.

In order to further characterize the two protein bands observed in SDS-PAGE, we have employed Capillary Electrophoresis analytical techniques coupled with ESI (electrospray ionization)-ion trap mass spectrometry detection (CE-ESI-IT-MS). The Total Ion Current (TIC) CE-MS profile of the purified α-DG mucin-like domain, α-DG(316–484), is characterized by an intense wide peak in a migration window within a 10.5-11.2 min time range, containing a small unresolved peak at the front (panel I). The deconvolution of the ESI mass spectrum registered between 10.5 and 11.2 min (panel III) revealed the presence of two protein species in the main peak, corresponding to the full-length α-DG(316–484) (theoretical mw 18252 Da) and to its truncated form devoid of the C-terminal fragment (theoretical mw 15470 Da), confirming the occurrence of a proteolytic cleavage. To prevent possible degradation due to dilution, a very concentrated sample was analyzed, which produced the loss of resolution power that usually characterizes CE separations. The analysis of the ESI mass spectrum registered between 9.8 and 10.5 min (small front peak A 2529.6) also enabled identification of the shed C-terminal fragment (theoretical mw 2529.9 Da). The panel II in Figure 2 shows the relative deconvoluted ESI mass spectrum.

**Figure 3:**
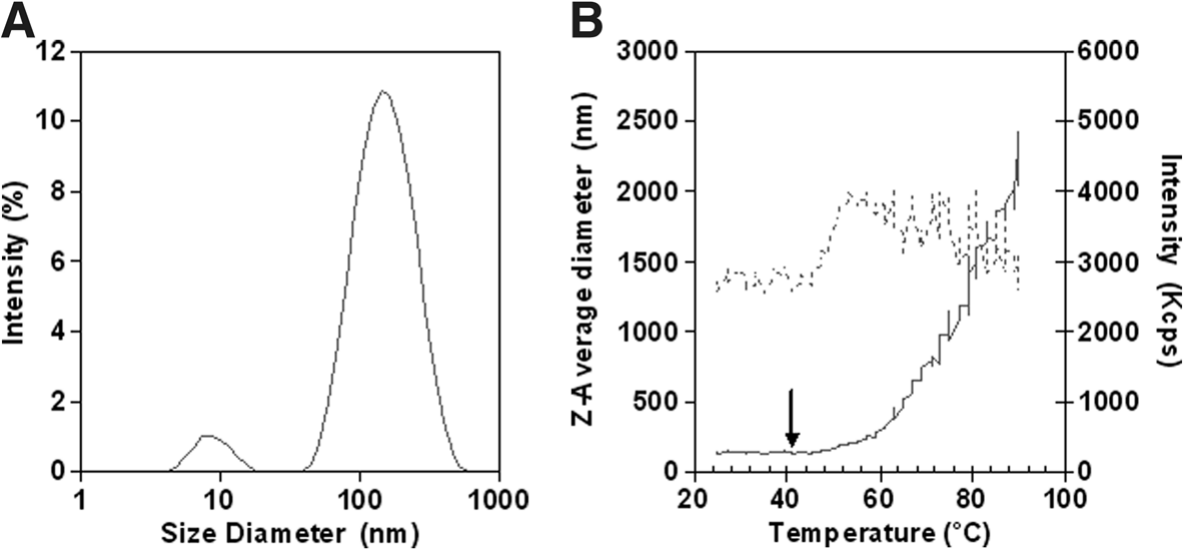
**Dynamic light scattering. A**) Intensity peak distribution and **B**) Z-Average diameter (solid line) and scattered intensity (dotted line) of α-DG(316–484) as function of temperature in PBS, pH 7.4. The arrow indicates the measured melting point.

To evaluate the stability of the mucin-like peptide, we have analyzed its behavior by dynamic light scattering (DLS) as a function of temperature. In Table 1, the Z-average (referring to the particle diameter) and polydispersity index (PdI), a measure of the distribution of the molecular masses, as well as the corresponding peak size, intensity (%) and mass (%) values are reported; the intensity peak distribution is also shown in Figure 3A. The experimental diameter of the full length α-DG(316–484), associated to the lower peak size (9 ± 3 nm), was similar to the theoretical one, calculated on the basis of its molecular weight (18 kDa). The presence of aggregates, represented by the higher peak of Figure 3A, accounts for only 1% of the total mass contribution to the scattered light of the protein solution.

**Table 1:** Dynamic light scattering of the recombinant mucin-like domain of human α-dystroglycan

	**Mode ± SD (nm)**	**PdI (%)**	**Intensity (%)**	**Mass (%)**
Z-average	111 ± 62	56	-	-
Peak 1	9 ± 3	28	5	99
Peak 2	142 ± 78	48	95	1

The protein melting point is defined as the temperature at which the protein is fully denatured. The change in size that accompanies the protein denaturation is identified using DLS techniques. Figure 3B shows the temperature dependent Z-average diameter and scattered intensity of α-DG(316–484) in phosphate buffered saline (PBS). Although α-DG(316–484) lacks secondary structure elements, as estimated by submitting its primary structure to several different softwares available for secondary structure prediction (data not shown), the observation that the Z-average diameter and scattering intensity values remain constant at temperatures lower than 40°C suggests the presence of a defined conformation of the protein; above this temperature the mean dimension of the protein increases exponentially, thus indicating the formation of denatured aggregates.

**Figure 4:**
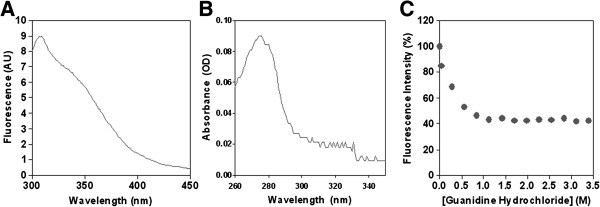
**Fluorescence and absorption spectra. A**) Fluorescence emission (Ex_λ_ = 280 nm) and **B**) absorbance spectra of α-DG(316–484) in PBS, pH 7.4. **C**) Effect of raising concentrations of guanidine hydrochloride on the fluorescence intensity of α-DG(316–484).

The fluorescence emission at 280 nm and the absorbance spectra of α-DG(316–484) are reported in Figure 4A and B. Lacking tryptophan and phenylalanine residues, the intensities measured were those of the single tyrosine residue in position 410 (see Additional file 1: Table S1), with an absorbance and fluorescence maximum at 274 and 307 nm, respectively.

Finally, the change in fluorescence intensity of α-DG(316–484) as a function of guanidine hydrochloride concentration is reported in Figure 4C. The protein shows an early decrease of fluorescence intensity upon unfolding, and the process seems to be already concluded at low guanidine concentration (1 M), thus confirming the unstable nature of the domain.

Overall, both DLS and the observed fluorescence behavior are in line with the idea that the recombinant mucin-like domain still possesses some sort of defined conformation at room temperature, although displaying a low stability as such conformation can be rapidly lost when increasing the temperature and/or the chaotropic agent concentration.

**Figure 5:**
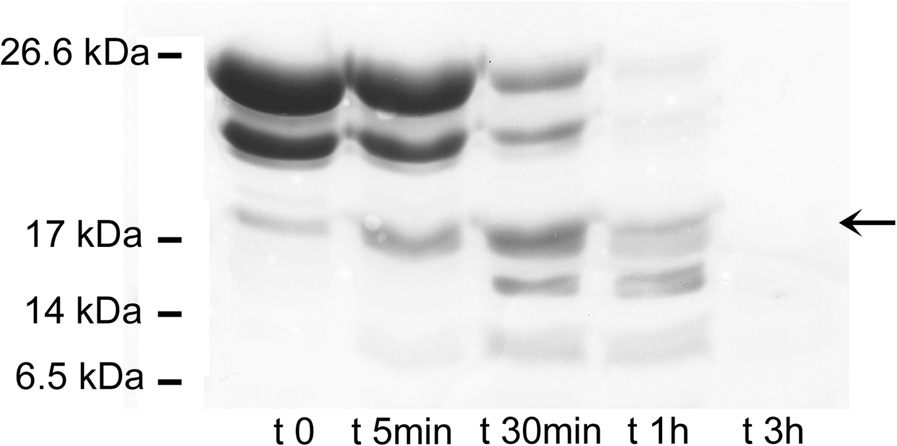
**Limited proteolysis of the recombinant**α**-DG mucin-like domain,**α**-DG(316–484).** Limited proteolysis of the α-DG(316–484) peptide (~5-10 μM) was carried out with 20 nM trypsin in PBS, pH 7.4 at room temperature. Aliquots of the reaction mix were collected at increasing times and analyzed by SDS-PAGE. Before incubation with trypsin (t 0), α-DG(316–484) already displays two bands corresponding to the entire peptide, α-DG(316–484), and to the slightly smaller truncated peptide α-DG(316–461), characterized by CE-ESI MS and originated via a C-terminal breakdown event. Noteworthy, the electrophoretic mobility of the whole α-DG(316–484) is much lower than expected on the basis of its molecular mass (18252 Da). Trypsin digests both the peptides producing one lower band at about 18 kDa (arrow), which is further fragmented, and completely degrades them in about 1 h.

Moreover, the reduced stability of the mucin-like peptide has been also confirmed by limited proteolysis at room temperature. Trypsin-based limited proteolysis of the mucin-like domain completely disrupts the protein in one hour (Figure 5), in a fashion that is reminiscent of what we have observed for the natively unfolded N-terminal β-DG domain [15]. It should be noted that typically stable proteins, including the C-terminal domain of α-dystroglycan, are resistant to limited proteolysis for up to 3 hours or longer [15].

## Conclusions

The data reported in this paper show that the recombinant mucin-like domain of α-DG, which we have produced in a non-glycosylated form, although quite unstable can be expressed and purified in reasonable amounts. Our results seem to be in line with what previously observed with ovine submaxillary mucin, in which the apomucin form collapses from an extended conformation when O-GalNAc O-glycosylation is removed [16].

Although our unglycosylated mucin-like peptide is clearly different from hypoglycosylated α-DG found in patients, may our results add some new insight into the current molecular view of dystroglycanopathies?

In muscles affected by dystroglycanopathies, α-DG is still localized at the sarcolemma, due to the non-covalent and sugar-independent interaction with the β-subunit [17,18]. The current molecular view of dystroglycanopathies implies that the observed reduction in sarcolemma stability would originate from the reduction of the binding affinity displayed by α-DG towards laminin, an event whose molecular basis is represented by an alteration of the O-mannosylated α-DG moieties and not by its dramatic hypoglycosylation. In addition, no evidence have been collected so far on the fact that the hypoglycosylated α-DG isoform(s) found in patients affected by dystroglycanopathies would be significantly destabilized and therefore exposed to preferential proteolysis. Of course, studies aiming at the analysis of the biochemical characteristics and stability of these hypoglycosylated α-DG molecules are warranted in order to further clarify this interesting point.

However, surely an extreme, but fascinating, view emerging from the data herein reported, is that α-DG glycosylation would be required primarily for stabilizing the conformation of its mucin-like domain (and conferring resistance to proteases like metalloproteases or others), as also proposed by Tran and colleagues [11], and secondarily for laminin binding. Although in the dystroglycanopathies currently known it is more common to observe a reduction of some specific O-Man (important for laminin binding) rather than of O-GalNAc (typical of the mucin-like portion) glycans, it is not unreasonable to foresee still unidentified cases of dystroglycanopathy in which a scenario would emerge of a significantly reduced level of glycosylation and consequent destabilization of the hypoglycosylated α-DG molecule.

Interestingly, 20 years ago protease inhibitors (Bestatin and Loxistatin) were already tested in Japan for their potential beneficial effect on Duchenne mice (*mdx*) and patients with some encouraging results [19]. A similar action has been recently observed using Batimastat (BB-94) [20]. Perhaps, in the future these drugs might be also tried more specifically on mouse models or patients affected by dystroglycanopathies, although a dangerous undesired effect of these protease inhibitors could be to inhibit the furin-driven proteolysis of the N-terminal domain of α-DG that seems to represent a necessary step along its maturation pathway [21].

Last but not least, from an exquisitely experimental point of view, our novel recombinant peptide may represent an interesting target to be employed to test *in vitro* the enzymatic action of POMT1 and POMT2, the first known players along the α-DG glycosylation cascade [22,23].

## Methods

### DNA manipulation

cDNA of human DG, cloned in the retroviral expression vector pBMN-IRES-PURO [24], was used as a template to generate by PCR the DNA construct corresponding to the mucin-like domain of α-DG, α-DG(316-484). Appropriate primers were used to amplify the DNA sequence of interest: forward 5′-CCC**GTCGAC**GCTACACCCACACCTGTCACTGCC-3′ and reverse 5′-CCC**GAATTC**TTAGGTGGTGGTGCGAATACGAGTAGG-3′ *SalI* and *EcoRI* restriction sites are in bold type.

### Protein expression and purification

The DNA construct obtained was purified and cloned into a bacterial vector appropriate to express the protein as a thioredoxin fusion product, also containing an N-terminal 6His tag and a thrombin cleavage site. The recombinant fusion protein was expressed in *E. coli* BL21(DE3) Codon Plus RIL cells and purified by affinity chromatography using a Ni-NTA resin (Novagen, USA). About 25 mg of thioredoxin fusion protein, previously dialyzed in a buffer containing 20 mM Tris–HCl, 0.15 M NaCl, 2.5 mM CaCl_2_, pH 8.4, were incubated with 8 NHI units of thrombin from human plasma (Sigma-Aldrich, USA) for 4 h at room temperature, in order to separate the mucin-like domain from its thioredoxin fusion partner. A second Ni-NTA affinity chromatography step was carried out to remove the thioredoxin fusion partner; the purity of the isolated mucin-like domain was checked by 12% SDS-PAGE. Protein staining in the polyacrylamide gel was performed with Coomassie Brilliant Blue R-250 dye.

### Limited proteolysis

Limited proteolysis of the mucin-like domain (~5-10 μM) was carried out with 20 nM trypsin in PBS, pH 7.4 at room temperature. Aliquots of the reaction mix were collected at 0, 5, 30, 60 and 180 min, and analyzed by 12% SDS-PAGE.

### Capillary Electrophoresis-mass spectrometry (CE-MS) experiments

The organic solvents used for CE-MS analysis were of LC-MS grade. Methanol, trifluoroacetic acid (TFA) (HPLC) and formic acid (98%) were from Mallinckrodt Baker B.V. (Deventer, The Netherlands). Ultra pure water was obtained from P.Nix Power System apparatus (Human, Seoul, Korea). Sodium hydroxide pellets, pro analysis, was from Merck (Darmstadt, Germany).

Capillary Electrophoresis experiments were performed on an Agilent Technologies (Waldbronn, Germany) automated apparatus, equipped with UV-diode array detector and external nitrogen pressure, and coupled to an Esquire 3000 plus mass spectrometer (Bruker Daltonics, Bremen, Germany) via a coaxial sheath liquid electrospray ionization (ESI) interface (Agilent Technologies, Waldbronn, Germany). The sheath liquid was composed by a mixture of water/methanol (30:70, v/v) containing 0.1% of formic acid (final content) and delivered by an external syringe pump (Cole Palmer, Vernon Hills, Illinois, USA) at a constant flow rate of 180 μl/h. Nebulising and drying gas (nitrogen) were set at 10 psi and 4 l/min, respectively. The nebulizer was set at 6 psi during sample injection process. The dry gas temperature was 250°C. The mass spectrometry capillary voltage was 4000 V. Mass spectrometry detection in ESI positive ionization was performed in full scan mode of acquisition using a 500–1500 m/z scan range, with a maximum accumulation time of 200 ms and upon activating the ICC instrumental function. Smart Parameters Setting (SPS) was set at 800 m/z of target mass value. Deconvolution of averaged ESI mass spectra was performed by MagTran 1.0 software.

Separations were performed using 1.0 M formic acid aqueous solution as background electrolyte (BGE) and a 50 μm I.D., 375 μm O.D. fused silica uncoated capillary (Composite Metal Services, Hallow, Worcs., UK) of total length of 85 cm. Effective lengths were 21.5 and 87 cm for the UV and MS detection, respectively. The temperature of the CE-MS assembly cartridge was 25°C and the applied CE running voltage 26 kV (positive polarity mode). Samples were injected at the anodic end of the capillary at 50 mbar × 10 sec followed by BGE injection at 50 mbar × 15 sec.

The sample was thawed at room temperature and immediately injected into the CE-MS apparatus after dilution 1:2 (v/v) in a TFA aqueous solution 0.1% (v/v).

### Dynamic light scattering

Dynamic light scattering (DLS) measurements on α-DG(316–484) protein solutions were carried out with a Zetasizer Nano S (Malvern Instruments, Malvern, U.K.) equipped with a 4 mW He-Ne laser (633 nm). Measurements were performed at 25.0 ± 0.1°C, at an angle of 173° from the incident beam. The sample was manually injected into a 3 mm path length Suprasil quartz cuvette (45 μl) (Hellma Italia, Milan, Italy); measurements were always performed after automatic instrument optical alignment and data collected for at least 15 minutes after injection. Each measurement consists of a subset of runs automatically determined, each being averaged for 10 seconds. The Z-average diameter, directly referring to the particle size, and the polydispersity index (PdI) were obtained from a Cumulants analysis of the measured intensity autocorrelation function, using version 6.32 of the Nano software. Measured size, intensity and mass distribution were also collected. Finally, the protein melting point was obtained measuring the temperature dependence of the Z-average diameter and scattering intensity of the protein solution, collecting data with a 2°C increment temperature ramp and 3 minute equilibrium time from 25 to 90°C.

### Fluorescence and absorbance studies

Fluorescence emission (Ex_λ_ = 280 nm) and absorbance spectra were recorded at 25°C in a 3 mm quartz cell, on samples dissolved in 100 mM PBS, pH 7.4 using a Spex (Edison, NJ, USA) FluoroMax spectrofluorimeter and a DU Series 700 UV/Vis spectrophotometer (Beckman Coulter, Fullerton, CA), respectively. In order to test the chemical stability of the α-DG(316–484) domain to unfolding, fluorescence spectra were recorded as a function of guanidine hydrochloride concentration as described elsewhere [25,26]. After each addition of denaturing agent, 10 min were allowed for the solution to reach equilibrium before spectra acquisition.

## Abbreviations

DG: Dystroglycan; DLS: Dynamic light scattering; PdI: Polydispersity index; CE: Capillary electrophoresis; TIC: Total ion current; EIC: Extracted ion current; IT-MS: Ion trap mass spectrometry; ESI: Electrospray ionization; TFA: Trifluoroacetic acid; PBS: Phosphate buffer saline; SDS-PAGE: Sodium dodecyl sulfate polyacrylamide gel electrophoresis; Ni-NTA: Nickel-nitrilotriacetic acid.

## Competing interests

The authors declare that they have no competing interests.

## Authors’ contributions

MB prepared the construct, carried out purification and limited proteolysis and participated in writing the manuscript; EDS carried out fluorescence and participated in writing the manuscript; GLS carried out dynamic light scattering; CD and CM carried out mass spectrometry; FS and BG revised the manuscript; AB conceived and directed the project, contributed to the experimental design of the study and wrote the manuscript. All authors read and approved the final manuscript.

## Supplementary Material

Additional file 1: Table S1 Primary structure of the recombinant mucin-like domain of human α-dystroglycan. Click here for file
