# Supplementary material for: Probing the stability of the “naked” mucin-like domain of human α-dystroglycan
Source: BMC Biochem. 2013 Jul 1;14:15. doi: 10.1186/1471-2091-14-15 (PMC3704865; doi:10.1186/1471-2091-14-15)
Supplement: Additional file 1: Table S1 — Primary structure of the recombinant mucin-like domain of human α-dystroglycan. [file 1471-2091-14-15-S1.doc]

**Table 1**

Primary structure of the recombinant mucin-like domain of human α-dystroglycan

**α-DG(316-484)**

*GSRVD***^316^**A**T**P**T**PVTAIGPP**TT**AIQEPPSRIVP**T**P**TS**PAIAPP**T**E**T**MAPPVRD

PVPGKP**T**V**T**IRTRGAIIQ**T**P**T**LGPIQP**T**RVSEAGTTVPGQIRPTMTIPG**Y**VEPTAVATPP

TTTTKKPRVSTPKPATPSTDSTTTTTRRPTKKPR**T**PRPVP**RVTTKVSITRLETASPPTRI**

**RTTT^484^**

**Number of amino acids:** 174

**Molecular weight:** 18252.0

**Theoretical pI:** 11.79

**Color code**

Red Italics: foreign residues (added for cloning purposes).

Bold Underlined Orange: the only aromatic residue, Tyr, in position 410 confers a typical fluorescence signal to the mucin-like peptide.

Bold Blue: Thr residues found as glycated by [9] and [10]; Ser residues found as glycated by [10].

Bold Red: fragment produced upon cleavage at the ^461^RV^462^ identified in this work.

Bold Underlined Black: Arg residue in position 461, representing the sensitive site to cleavage (occurring at its right side) that we have identified. PeptideCutter available at [www.expasy.org](http://www.expasy.org/) suggests that trypsin, thermolysin, clostripain and Arg-C proteinase (or their analogues present in *E. coli*) would be able to cut.
